# Marital status, widowhood duration, gender and health outcomes: a cross-sectional study among older adults in India

**DOI:** 10.1186/s12889-016-3682-9

**Published:** 2016-09-30

**Authors:** Jessica M. Perkins, Hwa-young Lee, K. S. James, Juhwan Oh, Aditi Krishna, Jongho Heo, Jong-koo Lee, S. V. Subramanian

**Affiliations:** 1 Harvard Center for Population and Development Studies, Harvard T.H. Chan School of Public Health, Boston, MA USA; 2 Massachusetts Center for Global Health, Massachusetts General Hospital, Boston, MA USA; 3 JW LEE Center for Global Medicine, Seoul National University College of Medicine, 71 Ihwajang-gil, Jongno-gu, Seoul, 110-810 Korea; 4 Jawaharlal Nehru University, New Delhi, India; 5 Department of Social and Behavioral Sciences, Harvard T.H. Chan School of Public Health, Boston, MA USA; 6 Public Health Joint Doctoral Program, San Diego State University & University of California, San Diego, CA USA; 7 Department of Family Medicine, Seoul National University College of Medicine, Seoul, Republic of Korea

**Keywords:** Widowhood, Aging, India, Gender, Self-rated health, Chronic disease, Cognition, Psychological distress

## Abstract

**Background:**

Previous research has demonstrated health benefits of marriage and the potential for worse outcomes during widowhood in some populations. However, few studies have assessed the relevance of widowhood and widowhood duration to a variety of health-related outcomes and chronic diseases among older adults in India, and even fewer have examined these relationships stratified by gender.

**Methods:**

Using a cross-sectional representative sample of 9,615 adults aged 60 years or older from 7 states in diverse regions of India, we examine the relationship between widowhood and self-rated health, psychological distress, cognitive ability, and four chronic diseases before and after adjusting for demographic characteristics, socioeconomic status, living with children, and rural–urban location for men and women, separately. We then assess these associations when widowhood accounts for duration.

**Results:**

Being widowed as opposed to married was associated with worse health outcomes for women after adjusting for other explanatory factors. Widowhood in general was not associated with any outcomes for men except for cognitive ability, though men who were widowed within 0–4 years were at greater risk for diabetes compared to married men. Moreover, recently widowed women and women who were widowed long-term were more likely to experience psychological distress, worse self-rated health, and hypertension, even after adjusting for other explanatory variables, whereas women widowed 5–9 years were not, compared to married women.

**Conclusions:**

Gender, the duration of widowhood, and type of outcome are each relevant pieces of information when assessing the potential for widowhood to negatively impact health. Future research should explore how the mechanisms linking widowhood to health vary over the course of widowhood. Incorporating information about marital relationships into the design of intervention programs may help better target potential beneficiaries among older adults in India.

**Electronic supplementary material:**

The online version of this article (doi:10.1186/s12889-016-3682-9) contains supplementary material, which is available to authorized users.

## Background

Empirical research spanning several decades has demonstrated that married people experience a range of physical and mental health benefits and greater functionality, self-rated health, and longevity as compared to non-married individuals [1–6]. Previous research exploring mechanisms linking marital status and health outcomes has posited several ways that marriage and health are causally associated [1, 7, 8]. First, marriage may offer economic, social, and psychological benefits, which may promote good health. These mechanisms may include access to sufficient economic resources, social control of behaviors by one’s spouse, or a sense of social support within the marital relationship. Second, transitioning to widowhood may induce significant strain upon a sudden change in resources, a change which leads to negative effects [9]. Alternatively, assortative mating based on health may occur [10, 11]. Also, research has found that healthier people tend to get married and stay married while unhealthy people tend to become widowed or divorced [12–16]. Regardless of mechanism, longitudinal studies have provided evidence of links between earlier marital status states and marital transitions to later wellbeing, health-related outcomes, chronic disease and mortality, [2, 7, 17–24] though the direction and strength of associations vary across studies and outcomes. Moreover, associations between marital status and health-related outcomes have remained even after adjusting for various sets of demographic and socioeconomic characteristics.

Widowhood is inherently a gendered and cultured experience as the salience of different mechanisms linking widowhood to health may depend on gender and on local norms [9]. Much of the formative research on marital status and health associations has been conducted in high-income countries where a substantial number of studies examining gender differences in the widowhood-health relationship have found evidence of worse outcomes for men, [5, 25–29] findings which are posited to be due to the loss of social and psychological support from the wife. However, results are mixed across the literature [30–33]. Moreover, research has provided evidence of variation in the relationships between marital status and health outcomes across cultures [9, 34–44]. Indeed, widowhood may differently affect men and women across contexts due to differences in gender norms and marriage traditions. For example, in some contexts widowhood may lead to increased financial strain for women while it may lead to increased household strain for men [45]. That relationship may differ in other contexts where roles and responsibilities differ by gender. Moreover, in patriarchal cultures, remarriage may not be a realistic option for women (particularly older women), thus forcing older women to remain widowed and without resources indefinitely [46]. In contrast, men may easily seek remarriage [44, 47]. If a woman is widowed from a young age without much ability to remarry due to cultural barriers (particularly if she already has children), then she may be economically disadvantaged for life. Alternatively, older men in some cultures where wives traditionally take care of men may be less able to cope with a loss of a spouse for longer periods whereas the existence of strong family ties (particularly other female familial relationships) may prevent negative effects in the short-term. In places where paternalistic norms are pervasive in everyday life (particularly in patterns of behavior related to economic opportunities, social activities, marriage traditions, and reputations), becoming widowed may severely restrict an individual’s ability to access financial, affective, informational, or physical resources, which in turn might affect health outcomes.

In India, a country with strict gender norms and traditional kinship systems, [48–50] widowhood is considered to be a dreaded phase of life among some groups, particularly for women [51]. Traditionally, the woman’s main role in India was to care for her husband. Upon losing her husband, the main purpose to life was lost. As she belonged to her husband’s family, in-laws frequent viewed widowed women as a burden. In the past, a traditional Hindu custom (which is the dominant religion in India) called for widows to commit suicide upon the death of their husband, [52] and although the practice is illegal now, it is still occurs (though obviously with lower frequency). More recently, the ‘city of widows’ in India has been highlighted, which is a holy site that is home to thousands of widowed women who live in dire circumstances and beg for money [53, 54]. In general, widowhood for women in India is a very tenuous period of life, highlighted by significant poverty, lack of social support, a lack of ability to remarry, and a greater risk of mortality [46, 55–57]. Widowhood for elderly women in India may be a highly stigmatizing and potentially public experience as, according to traditional customs, they may shave their heads, wear only plain or white clothing, eat only two or fewer meals per day, and not be permitted to attend social gatherings or to re-marry [58–60]. Thus, given historical precedent and India’s patriarchal society embodying strict norms, attitudes, and practices that typically affect the social status of the elderly, and women in particular, [61] widowed older women in India may face significant discrimination (experienced or perceived) as well as a lack of economic resources [51, 62–64]. These issues may in turn affect health outcomes. In this context, widowhood may present substantial disadvantages for women if the transition signifies a loss of resources, particularly in the long-term, though there may be differences by socioeconomic status and other demographic factors, as well as by region [65–67]. In contrast, widowhood may not be associated with health outcomes for men if other women in the family immediately take over the daily household chores and any care the widowed men may need.

Most studies examining health-related outcomes as a function of marital status among older adults in India have found worse health to be associated with widowed status as compared to married status [68–73]. These studies, however, adjusted for varying sets of covariates and many of the studies only focused on self-rated health as the outcome. Moreover, few studies have focused on the potential health effects of widowhood for men in India. Yet, as the aging population of India increases in a context where access to and affordability of social services is limited for older individuals, [74, 75] it is important to identify individuals who are more at risk for worse health outcomes among the general older adult population. Being widowed represents a relatively easy marker.

Thus, assessing whether there is evidence of a direct relationship between widowhood and multiple subjective and objective health-related outcomes and chronic diseases among older adults in India after adjusting for a large set of demographic and socioeconomic factors (such as caste, education, wealth, religion, living with children, rural/urban location, etc.) is warranted. Moreover, examining these associations separately for men and women is critical due to unequal gender norms in India and also because a higher proportion of men in India remarry while an increasing fraction of women remain widows [70]. Finally, no studies of which we are aware have examined how duration of widowhood is associated with outcomes among older adults in India. Yet, men and women recently widowed may experience worse outcomes than people widowed for much longer. For example, men who are more recently widowed may experience stressful transitions and immediate loss of a known daily support. In the long run, however, they are likely well-cared for by other female relatives. Alternatively, women who have been widowed for a long time may be the worst off due to long-term reduced access to resources and, perhaps, poor treatment by their husband’s family. Previous studies from other countries have revealed a relationship between duration widowhood and self-reported health, psychological wellbeing, or other health outcomes, [20, 26, 37, 76–78] though findings have differed across populations and outcomes.

The current study attempts to address these gaps in the literature by providing empirically descriptive answers to two questions: First, to what extent is widowhood associated with a variety of health-related outcomes and chronic diseases among older men and women, separately, in India, after adjusting for several demographic and socioeconomic indicators? Second, is there evidence that widowhood duration matters in these relationships? We hypothesized that being widowed (without regards to duration) would be associated with worse health outcomes for both men and women, even after adjusting for several indicators of socioeconomic status, living arrangement, and place, though we thought that the strength of the relationship would be greater among women. Moreover, we hypothesized that being widowed for longer would be associated with an even greater risk of poor health outcomes for women given a potential longer period of resource restriction.

## Methods

### Sample

We utilized a dataset called “Building Knowledge Base on Population Ageing in India”, [79] based on older adults aged 60 years or older from seven states in India (Himachal Pradesh, Kerala, Maharashtra, Odisha, Punjab, Tamil Nadu and West Bengal). These states were purposely chosen during the design of this study as they represented all regions of India and had a higher prevalence of elderly individuals as compared to the national average. In each state, participants were drawn from 40 rural and 40 urban Primary Sampling Units (PSUs), which were systematically sampled according to a probability proportional to population size. 16 households including at least one 60 + year old individual were sampled per PSU, creating a sample frame of 1,280 households. More detailed information on how households were selected can be found in the BKPAI report [80]. All household residents 60+ years old were eligible for the study.

**Fig. 1:**
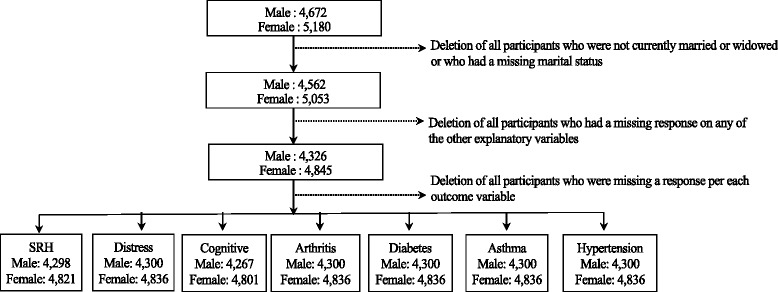
Flowchart of final analytical sample size for men and women included in this study

From May to September 2011, 8,329 household interviews were conducted in 560 PSUs (representing a 95 % household response rate) and 4,672 men and 5,180 women were individually interviewed (leading to a 93 % individual response rate). We only included adults who were either currently married or who were widowed (*n* = 9,615) as the sample sizes for women who were divorced, separated, cohabiting or never married were 1 % or less each. The final analytical samples for each outcome included respondents with no missing values across explanatory variables or the outcome. Figure 1 provides a flowchart of the final analytical sample sizes and the number of participants excluded. We chose to exclude individuals with missing data rather than impute values for missing responses as there were relatively little missing data. As the data used for this work were completely de-identified and publically available for secondary analysis, the first author’s institutional review board approved this study and deemed it to be exempt from full institutional review.

### Outcomes

To capture self-rated health, respondents were asked to rate their current health status on a 5-point scale where 1 = excellent, 2 = very good, 3 = good, 4 = fair, and 5 = poor. The order was reversed for ease of interpretation with a value of 1 for poor health and a value of 5 for excellent health. A binary variable representing ‘poor health’ was also created where the responses fair and poor = 1 and excellent, very good, and good = 0. Psychological distress was measured using the General Health Questionnaire composed of 12 items [81, 82]. This tool has been widely used in mental health research and previous studies have demonstrated its validity and usefulness in several contexts, including India [83–85]. The items ask whether the respondent has recently experienced a particular stressful symptom or behavior. Each item was rated on a 4-point scale (0 = “less than usual”, 1 = “no more than usual”, 2 = “rather more than usual”, or 3 = “much more than usual”). Items were rescored using 0-0-1-1 responses as other studies have previously done. The items were then summed together with a total possible score ranging from 0 to 12, which was treated as continuous variable. A higher score indicated a greater degree of psychological distress. Probable ‘common mental disorder’ was derived from the psychological distress score by creating a dichotomous variable using a cutoff of 5 or less vs. 6 or higher with the latter indicating a probable common mental disorder’ [86]. Immediate recall of words was used to measure cognitive ability [87]. A list of 10 commonly used words was read out to the respondents, who were then asked to recall the words within two minutes. The number of words recalled (0 to 10) was recorded. Therefore, a higher score represented better cognitive ability.

Four chronic morbidity outcomes were measured by asking, separately, whether the respondents had ever been told by a doctor or a nurse that he or she had high blood pressure (hypertension), diabetes, arthritis, or asthma (yes/no responses). A binary variable indicating whether a respondent had been told that he or she had one or more of those four diseases was created.

### Explanatory variables

For this study, marital status was indicated as either being currently married (reference group) or widowed. We also created a second marital status variable where the widowed category was split into three groups according to duration of widowhood: 0–4 years, 5–9 years, or 10+ years. The split between 4 and 5 years was based on previous rsearch showing differences between the more recently widowed and the longer-term widows, [37] and the split between 9 and 10 years was chosen because about half of people were widowed beyond that point. Age was divided into five-year intervals as 60–64 years (reference), 65–69 years, 70–74 years, 75–79 years, and 80+ years. Respondents indicated a caste (Scheduled Caste (reference), Scheduled Tribe, Other Backward Caste, and other caste), and whether they stayed with children in the same household (reference) or not. Completed education was categorized as none (reference), 1–5 years, 6–10 years, and 11 or more years. Work status was a binary variable categorized as having worked during the past one year versus not having worked. Household wealth quintiles were calculated using information on 30 assets and housing characteristics [50, 80]. Location of the household in a rural or urban location and the state was also recorded.

### Statistical analysis

Gender-stratified, multivariable, multilevel linear and logistic regression analyses were used to estimate the association between an outcome and widowhood (as compared to being married) while accounting for the clustering of observations at the PSU and district levels. Model 1 used the binary marital status variable and adjusted for age, caste, living with children, urban/rural location, and state. Model 2 adjusted for socioeconomic-based variables including education, work status, and household wealth in addition to the variables included in Model 1. Finally, Model 3 was equivalent to Model 2 except it utilized the marital status variable that was further categorized according to widowhood duration of less than 5 years, from 5 to 9 years, and 10 or more years.

## Results

Table 1 provides descriptive statistics about the sample and also the average scores of self-reported health, psychological distress, and cognitive ability across sub-categories of socio-demographic characteristics. For context, almost two-thirds of the sample population were within the ages of 60 to 69 years while about 10 % were 80 years or older. In addition, 4 % of men had been widowed for 0–4 years, 4 % for 5–9 years, and 6 % for 10 years or more. Among women, 14 % had been widowed for 0–4 years, 13 % for 5–9 years, and 34 % for 10 years or more. Table 2 provides the prevalence among the sample population of being diagnosed with each of four chronic diseases (hypertension, diabetes, arthritis, and asthma), being diagnosed with at least 1 chronic disease, being in poor health, and having a probable common mental disorder. Among men, 30 % who were currently married vs. 36 % who were widowed for 10 years or more had at least one chronic disease, 49 to 57 % of men in those same groups reported being in poor health, and 24 to 35 % had a probable mental disorder. Among women, the prevalence of being diagnosed with at least 1 chronic disease, being in poor health, and having a probable common mental disorder, separately, was higher among married women than the associated prevalence among women who had been widowed for 5 to 9 years.

**Table 1:** Descriptive characteristics of a representative sample of older adults (60 + years) across seven states in India in 2011 (*N* = 9,171)

	Total N and % in sub-category				Self-Rated Health				Psychological Distress				Cognitive Ability			
	Male		Female		Male		Female		Male		Female		Male		Female	
	N	%	N	%	Mean	S.D	Mean	S.D	Mean	S.D	Mean	S.D	Mean	S.D.	Mean	S.D.
Marital Status																
Currently Married	3698	85.5	1888	39	3.4	1.0	3.5	1.0	3.4	3.9	3.1	3.6	4.6	1.7	4.3	1.6
Widowed 0 to 4 years	189	4.4	690	14.2	3.7	0.9	3.7	1.0	3.7	3.8	4.3	3.9	4.1	1.9	3.8	1.6
Widowed 5 to 9 years	162	3.7	631	13	3.4	1.0	3.5	0.9	3.9	4.2	3.5	3.9	3.9	1.6	4.2	1.6
Widowed 10+ years	277	6.4	1636	33.8	3.6	1.0	3.8	1.0	3.0	3.6	4.5	3.9	3.9	1.7	3.5	1.6
Age Group																
60–64	1519	35.1	1716	35.4	3.2	1.0	3.4	1.0	2.6	3.4	3.0	3.5	4.9	1.6	4.4	1.6
65–69	1219	28.2	1324	27.3	3.4	1.0	3.6	1.0	3.0	3.6	3.6	3.8	4.6	1.6	4.0	1.6
70–74	771	17.8	819	16.9	3.4	1.1	3.7	1.0	3.5	3.7	4.4	4.0	4.3	1.6	3.7	1.5
75–79	395	9.1	462	9.6	3.6	1.0	3.8	1.0	3.5	3.8	4.5	3.8	4.0	1.8	3.4	1.6
80+ years	422	9.8	524	10.8	3.8	1.1	3.9	1.0	4.4	4.1	5.1	4.1	3.5	1.8	3.0	1.7
Living with Children																
No	1255	29	1328	27.4	3.4	1.0	3.6	1.0	3.2	3.8	3.8	3.9	4.6	1.7	4.0	1.7
Yes	3071	71	3517	72.6	3.4	1.0	3.6	1.0	3.1	3.6	3.7	3.8	4.4	1.7	3.9	1.6
Caste																
Scheduled Caste	850	19.6	968	20	3.6	1.0	3.7	1.0	3.6	3.9	4.3	4.0	4.2	1.7	3.6	1.5
Schedule Tribe	222	5.1	250	5.2	3.3	0.9	3.5	0.9	4.1	3.8	4.8	4.0	4.1	1.4	3.7	1.5
Other Backward Caste	1515	35	1702	35.1	3.5	1.0	3.6	1.0	3.5	3.9	4.0	3.9	4.5	1.6	4.0	1.7
None of them	1739	40.2	1925	39.7	3.3	1.1	3.5	1.1	2.4	3.3	3.2	3.6	4.6	1.8	4.0	1.7
Work status (in the last 1 year)																
No	2759	63.8	4334	89.5	3.5	1.0	3.6	1.0	3.3	3.8	3.7	3.8	4.4	1.8	3.9	1.7
Yes	1567	36.2	511	10.5	3.3	1.0	3.4	1.0	2.8	3.4	4.0	3.7	4.6	1.6	4.0	1.5
Education																
None	1353	31.3	2940	60.7	3.6	1.0	3.7	1.0	4.3	4.0	4.4	3.9	3.9	1.5	3.6	1.5
< 5 years	945	21.8	966	19.9	3.5	1.0	3.6	1.1	3.6	3.7	3.4	3.6	4.1	1.6	4.0	1.6
6 to 10 years	1406	32.5	723	14.9	3.3	1.0	3.4	1.0	2.2	3.2	2.1	3.0	4.9	1.6	4.8	1.6
≥ 11 years	622	14.4	216	4.5	3.0	1.1	3.3	1.1	1.8	2.8	1.9	3.0	5.5	1.8	5.4	1.9
Household Wealth Quintile																
Bottom	779	18	1000	20.6	3.7	0.9	3.8	0.9	5.0	3.7	5.4	3.8	3.9	1.4	3.6	1.5
Second	839	19.4	1007	20.8	3.5	1.0	3.6	1.0	4.0	3.9	4.4	3.9	4.1	1.6	3.7	1.5
Third	820	19	987	20.4	3.5	1.0	3.6	1.0	3.2	3.7	3.5	3.8	4.3	1.7	3.9	1.6
Fourth	934	21.6	904	18.7	3.3	1.0	3.5	1.0	2.3	3.4	3.0	3.7	4.8	1.7	4.2	1.7
Top	954	22.1	947	19.6	3.2	1.1	3.5	1.1	1.5	2.6	2.5	3.2	5.1	1.8	4.4	1.8
Place																
Urban	2035	47	2322	47.9	3.3	1.0	3.5	1.0	2.6	3.5	3.4	3.7	4.7	1.8	4.1	1.7
Rural	2291	53	2523	52.1	3.5	1.0	3.7	1.0	3.6	3.8	4.1	3.9	4.3	1.6	3.8	1.5
State																
Himachal Pradesh	727	16.8	709	14.6	3.1	1.1	3.3	1.1	1.8	3.3	2.8	3.9	5.2	1.8	4.2	1.7
Punjab	621	14.4	696	14.4	3.6	0.9	3.8	0.9	1.5	2.8	1.9	3.0	4.4	1.6	4.2	1.6
West Bengal	477	11	550	11.4	3.9	0.8	4.2	0.8	4.5	2.9	4.7	3.1	3.6	1.4	3.1	1.2
Orissa	703	16.3	713	14.7	3.3	0.9	3.4	0.9	4.3	3.6	5.1	3.7	4.1	1.5	3.7	1.5
Maharashtra	624	14.4	704	14.5	3.1	1.1	3.2	1.0	3.5	3.6	3.9	3.8	4.6	1.5	4.1	1.5
Kerala	539	12.5	736	15.2	3.7	1.2	3.9	1.1	1.9	2.9	3.2	3.6	4.0	1.9	3.7	1.9
Tamil Nadu	635	14.7	737	15.2	3.4	0.7	3.5	0.8	4.5	4.5	4.8	4.3	4.9	1.6	4.4	1.5

**Table 2:** Prevalence of chronic disease, poor health, and probable mental disorder among older adults across seven states in India in 2011 (unit = %)

	Hypertension		Diabetes		Asthma		Arthritis		At least 1 chronic disease		Poor Health		Probable Mental Disorder	
	Male	Female	Male	Female	Male	Female	Male	Female	Male	Female	Male	Female	Male	Female
Marital Status														
Currently Married	18	24	12	11	8	6	23	34	30	37	49	52	24	24
Widowed 0 to 4 years	20	29	16	12	11	7	24	30	31	36	62	65	28	38
Widowed 5 to 9 years	13	18	12	8	6	7	26	28	28	31	51	45	34	30
Widowed 10+ years	18	28	9	11	10	7	31	35	36	40	57	64	35	39
Age Group														
60–64	15	21	12	10	5	5	17	28	23	32	41	47	20	24
65–69	18	26	11	11	8	7	23	35	29	38	49	55	23	31
70–74	20	28	12	11	9	8	28	38	35	43	54	62	27	37
75–79	24	31	15	13	10	8	28	31	36	37	61	69	28	37
80+ years	22	27	11	8	15	9	38	39	45	44	64	74	38	46
Living with Children														
No	18	21	13	9	8	7	24	34	30	38	48	56	27	33
Yes	18	27	12	11	8	7	24	33	30	37	51	57	24	31
Caste														
Scheduled Caste	14	22	7	8	8	5	26	35	31	38	58	61	30	37
Schedule Tribe	10	13	5	4	7	8	25	30	28	31	43	47	31	38
Other Backward Caste	17	24	12	12	8	7	23	27	29	31	50	57	30	35
None of them	22	30	15	12	9	7	24	38	30	43	47	56	18	26
Work status (in the last 1 year)														
No	21	27	14	12	9	7	24	34	31	38	53	58	27	32
Yes	13	14	8	4	6	6	23	28	28	30	44	49	22	34
Education														
None	14	22	6	7	9	6	33	37	36	40	59	61	37	39
< 5 years	17	32	10	16	10	8	23	27	30	34	56	56	30	26
6 to 10 years	19	30	14	16	8	7	19	25	26	30	43	42	16	14
≥ 11 years	29	32	22	19	5	6	16	35	25	41	35	48	12	14
Household Wealth Quintile														
Bottom	9	13	3	4	8	5	27	31	29	34	60	64	44	49
Second	13	18	8	6	9	6	26	34	29	37	53	57	35	40
Third	17	28	9	12	9	7	26	31	31	35	49	56	24	29
Fourth	18	30	13	14	8	8	21	33	29	39	45	52	17	23
Top	31	38	24	20	7	8	21	36	32	42	44	55	9	17
Place														
Urban	20	28	15	13	7	7	21	31	28	36	46	53	20	29
Rural	17	23	9	8	9	7	27	35	32	39	54	60	30	35
State														
Himachal Pradesh	15	22	9	8	9	6	24	43	29	46	38	46	13	22
Punjab	25	38	13	14	7	6	38	52	44	56	60	68	10	13
West Bengal	19	28	9	7	3	2	15	25	25	31	71	80	37	43
Orissa	15	17	9	5	5	4	22	29	24	30	45	47	36	46
Maharashtra	13	16	9	6	13	11	31	36	33	37	38	41	26	31
Kerala	35	45	32	28	16	13	13	17	33	30	62	71	11	23
Tamil Nadu	9	12	6	6	3	3	20	27	23	30	45	50	42	46

Table 3 displays the estimated relationships between widowhood and linear health-related outcomes (scores of self-rated health, psychological distress, and cognitive ability, separately), as well as between widowhood and binary health-related outcomes (being in poor health and having a probable mental disorder, separately) and binary indicators of chronic disease (diagnosed with hypertension, diabetes, arthritis, asthma, and 1 or more chronic diseases, separately) for men and women separately. The relationships between widowhood and all outcomes except for diabetes, arthritis, asthma, and having 1 or more chronic diseases were statistically significant in Model 1 for women with widowhood being associated with worse outcomes. The same pattern was found for men, but only for cognitive ability and having a probable common mental disorder. Adjusting for socioeconomic factors in Model 2 attenuated the relationships between widowhood and outcomes though most of the estimates remained statistically significant and in the predicted direction. There was no evidence of widowhood acting as a protective factor for either gender.

**Table 3:** Linear and logistic regression estimates of the association between widowhood and health among older adults in India

		Men				Women			
		Model 1		Model 2		Model 1		Model 2	
Linear Outcome		b	(95 % CI)	b	(95 % CI)	b	(95 % CI)	b	(95 % CI)
Self-Rated Health (higher = worse)	Widowed (vs. married)	0.06	(−0.03, 0.14)	0.003	(−0.08, 0.08)	0.11***	(0.06, 0.17)	0.08**	(0.02, 0.14)
Psychological Distress (higher = worse)	Widowed (vs. married)	0.22	(−0.06, 0.50)	0.01	(−0.26, 0.29)	0.48***	(0.26, 0.69)	0.33**	(0.12, 0.55)
Cognitive Ability (higher = better)	Widowed (vs. married)	−0.30***	(−0.43, −0.16)	−0.18**	(−0.31, −0.05)	−0.22***	(−0.31, −0.13)	−0.11*	(−0.20, −0.02)
Binary Outcome		AOR	(95 % CI)	AOR	(95 % CI)	AOR	(95 % CI)	AOR	(95 % CI)
Being in Poor Health	Widowed (vs. married)	1.14	(0.94, 1.39)	1.02	(0.84, 1.25)	1.17*	(1.01, 1.35)	1.08	(0.93, 1.25)
Having a Probable Mental Disorder	Widowed (vs. married)	1.33*	(1.07, 1.66)	1.17	(0.93, 1.46)	1.38*	(1.18, 1.61)	1.25*	(1.07, 1.48)
Diagnosed with Hypertension	Widowed (vs. married)	0.86	(0.67, 1.10)	0.86	(0.67, 1.11)	1.21*	(1.04, 1.42)	1.29*	(1.10, 1.52)
Diagnosed with Diabetes	Widowed (vs. married)	1.15	(0.87, 1.54)	1.21	(0.90, 1.63)	1.03	(0.83, 1.28)	1.14	(0.91, 1.42)
Diagnosed with Asthma	Widowed (vs. married)	1.02	(0.74, 1.40)	0.96	(0.70, 1.32)	1.18	(0.90,1.54)	1.20	(0.92, 1.57)
Diagnosed with Arthritis	Widowed (vs. married)	0.93	(0.75, 1.16)	0.89	(0.72, 1.11)	1.07	(0.92,1.23)	1.07	(0.92, 1.24)
Diagnosed with at least 1 chronic disease	Widowed (vs. married)	0.88	(0.72, 1.08)	0.85	(0.70, 1.05)	1.11	(0.97, 1.28)	1.12	(0.97, 1.29)

* *p* < .05; ** *p* < .01; ***p < .001. *Notes*: Model 1 was adjusted for age, caste, living with children, urban/rural, and state. Model 2 was adjusted for all explanatory variables in Model 1 + education, work status, and household wealth quintile. For all models, estimates also accounted for survey design as a three-level (individual, primary sampling unit and district) random intercepts model was used

When the widowhood category of marital status was re-categorized by taking into account widowhood duration, estimates from Model 3 indicated that only some categories of widowhood were significantly different in terms of outcomes as compared to married individuals (Table 4). Among men, diabetes, cognitive ability, and having a probable common mental disorder, were associated with widowhood, but only for widowers of certain duration. For example, men who were widowed within 0–4 years were more likely to have been diagnosed with diabetes (AOR = 1.64, 95 % CI = 1.06 to 2.54); men who were widowed for 5–9 years were more likely to recall fewer words (b = −0.24, 95 % CI = −0.47 to −0.01) and, men who had been widowed for 10+ years were more likely to have a probable common mental disorder (AOR = 1.38, 95 % CI = 1.01 to 1.88).

**Table 4:** Linear and logistic regression estimates of the association between widowhood accounting for duration and health among older adults in India

		Model 3			
	Widowhood Status (vs. married as the reference)	Men		Women	
Linear Outcome		b	(95 % CI)	b	(95 % CI)
Self-Rated Health	Widowed 0 to 4 years	0.1	(−0.04, 0.23)	0.14**	(0.06, 0.22)
	Widowed 5 to 9 years	−0.12	(−0.26, 0.03)	−0.01	(−0.09, 0.08)
	Widowed 10+ years	0.01	(−0.11, 0.12)	0.09*	(0.02, 0.15)
Psychological Distress	Widowed 0 to 4 years	−0.22	(−0.67, 0.24)	0.51**	(0.21, 0.80)
	Widowed 5 to 9 years	−0.09	(−0.58, 0.40)	0.06	(−0.26, 0.37)
	Widowed 10+ years	0.23	(−0.16, 0.61)	0.37**	(0.12, 0.62)
Cognitive Ability	Widowed 0 to 4 years	−0.13	(−0.34, 0.09)	−0.13*	(−0.26, −0.01)
	Widowed 5 to 9 years	−0.24*	(−0.47, −0.01)	−0.01	(−0.14, 0.13)
	Widowed 10+ years	−0.18	(−0.37, 0.001)	−0.15**	(−0.25, −0.04)
Binary Outcome		AOR	(95 % CI)	AOR	(95 % CI)
Poor Health	Widowed 0 to 4 years	1.32	(0.94, 1.87)	1.40**	(1.13, 1.72)
	Widowed 5 to 9 years	0.82	(0.57, 1.17)	0.77*	(0.62, 0.95)
	Widowed 10+ years	0.99	(0.75, 1.31)	1.11	(0.93, 1.31)
Probable Mental Disorder	Widowed 0 to 4 years	0.92	(0.62, 1.36)	1.43**	(1.14, 1.78)
	Widowed 5 to 9 years	1.13	(0.76, 1.67)	1.09	(0.86, 1.38)
	Widowed 10+ years	1.38*	(1.01, 1.88)	1.25*	(1.04, 1.50)
Hypertension	Widowed 0 to 4 years	0.93	(0.62, 1.38)	1.52***	(1.22, 1.90)
	Widowed 5 to 9 years	0.7	(0.43, 1.14)	0.86	(0.67, 1.11)
	Widowed 10+ years	0.92	(0.65, 1.29)	1.39***	(1.16, 1.67)
Diabetes	Widowed 0 to 4 years	1.64**	(1.06, 2.54)	1.15	(0.85, 1.56)
	Widowed 5 to 9 years	1.36	(0.81, 2.30)	0.93	(0.66, 1.32)
	Widowed 10+ years	0.86	(0.54, 1.36)	1.23	(0.95, 1.58)
Arthritis	Widowed 0 to 4 years	0.73	(0.50, 1.07)	1.01	(0.82, 1.25)
	Widowed 5 to 9 years	0.97	(0.66, 1.44)	0.95	(0.76, 1.19)
	Widowed 10+ years	0.96	(0.71, 1.29)	1.15	(0.97, 1.36)
Asthma	Widowed 0 to 4 years	1.23	(0.75, 2.02)	1.32	(0.91, 1.90)
	Widowed 5 to 9 years	0.70	(0.36, 1.37)	1.37	(0.94, 2.00)
	Widowed 10+ years	0.93	(0.60, 1.45)	1.07	(0.79, 1.46)
Diagnosed with at least 1 chronic disease	Widowed 0 to 4 years	0.8	(0.56, 1.13)	1.12	(0.92, 1.37)
	Widowed 5 to 9 years	0.78	(0.54, 1.15)	0.95	(0.77, 1.18)
	Widowed 10+ years	0.93	(0.70, 1.23)	1.20*	(1.02, 1.41)

* *p* < .05; ** *p* < .01; ***p < .001. *Notes*: Model 3 adjusted for all explanatory variables in Model 2, but used a four-category marital status variable accounting for widowhood duration. Model 3 also accounted for survey design as a three-level (individual, primary sampling unit and district) random intercepts model was used

Among women, there was evidence of a role for duration for most outcomes (except for diabetes, and again arthritis and asthma). For example, women widowed for 4 years or less or for more than 10 years were more likely to report worse self-rated health (b = 0.14, 95 % CI = 0.06 to 0.22, and b = 0.09, 95 % CI = 0.02 to 0.15, respectively), and worse psychological distress (b = 0.51, 95 % CI = 0.21 to 0.80, and b = 0.37, 95 % CI = 0.12 to 0.62, respectively), as well as recall fewer words (b = −0.13, 95 % CI = −0.26 to −0.01, and b = −0.15, 95 % CI = −0.25 to −0.04, respectively) than married women, separately. In addition, hypertension was more likely among women who were recently widowed or who were widowed for a long time (AOR = 1.52, 95 % CI = 1.22 to 1.90, and AOR = 1.39, 95 % CI - 1.16 to 1.67, respectively). Overall, results indicated that mostly recently widowed women and long-term widowed women were at risk for worse health outcomes compared to married women whereas women who were widowed for 5–9 years were no different than women who were married.

Additional file 1: Tables S1–S4 provide the estimates for the relationships between the other explanatory variables and outcomes. Age was a strong predictor for all outcomes for both genders except for diabetes. Men and women with higher education and higher wealth status were more likely to have better health-related outcomes and lower odds of experiencing chronic disease. Wealth status showed no association with arthritis, asthma, or having one or more chronic diseases. Living with children was not associated with any of the outcomes for either men or women (Additional file 1: Tables S1-S4).

## Discussion

This analysis of marital status and health-related outcomes and chronic diseases among older adults across India suggests that, for women, widowhood (as opposed to being married) may be a risk factor for poor self-rated health, psychological distress and reduced cognitive ability, as well as having a probable common mental disorder and being diagnosed with hypertension, separately. There is no evidence of these associations among men except for with cognitive ability and having a probable common mental disorder. Results did not substantively change for men or women even after adjusting for several demographic and socioeconomic factors. Moreover, examining these associations through a widowhood duration lens provides evidence that the relationship between widowhood and health outcomes may be more nuanced than a simple binary effect (widowed vs. not widowed). For women, being widowed for a short amount of time or for the long-term seemed to be worse for many health outcomes as compared to married women. In contrast, the relevance of widowhood duration varied across health outcomes for men though the health of married men was, for the most part, no different than the health of widowed men regardless of widowhood duration.

More recently widowed older women in India may struggle to cope with new substantial losses in access to financial resources and a new (often diminished) social role within their in-laws or son’s household, which may negatively affect their health. Not only may women lose regular economic support when transitioning to widowhood, they may also be deprived of any inheritance rights and lose overall purpose within the household. In contrast, the health of women widowed for an intermediate amount of time (e.g. 5 to 9 years) may not differ from the health of married women because these widowed women have been able to cope (at least temporarily) with the passing of their spouse, have settled into a new household context, and are not yet facing the psychological prospect of having to live for many years without a new spouse nor having to yet address the long-term issues of not having access to resources that a spouse would provide. Perhaps they have found a way to survive by building new social ties or have taken on new responsibilities within their husband’s or son’s family. In addition, survival selection could be playing a role; women who survived to be widowed 5 to 9 years may, on average, be healthier than the same cohort of women when they had only been widowed 4 years or less as the unhealthiest women in that cohort may have died by the time this cohort of women became widowed for 5 to 9 years. Finally, the health of women who have been widowed for 10 or more years may have once again simply deteriorated in contrast to married women perhaps because they have both psychologically and physically remained without resources and spousal support for a decade, a situation which would likely continue until they pass away (as they are not likely to remarry). Critically, regardless of duration, older widowed Indian women may face an interwoven set of losses and challenges that affect their health outcomes [88].

Among men, the situation appears much more varied. For the most part in India, men’s access to resources does not change when they become widowed. Instead of marital status or duration of widowhood, marital quality might be a better predictor of health outcomes for men. Interestingly, however, more recently widowed men may be susceptible to diabetes-related risk factors, such as a change in diet as wives in India are typically responsible for household chores, including cooking. The death of a wife might lead to a worse diet and onset of diabetes at the beginning before another woman takes over regular preparation of food for the newly widowed man. In contrast, men who would have remained widowed for a long time might have already died or remarried (due to finding it too difficult) whereas the men who remain single long after being widowed are perhaps the most resilient. Importantly, the pathways through which health outcomes are affected at different points during widowhood for both men and women warrant further exploration.

Our findings about self-rated health are similar to previous studies in China and India [37, 70, 73]. In addition, our findings suggesting reduced cognitive ability among widowed men are similar to a study conducted in three countries in Europe [17]. However, the present results indicating a negative relationship between widowhood and a number of health outcomes for women and a lack of a relationship between widowhood and most health outcomes for widowed men are inconsistent with many studies from high-income countries, which have typically found a marriage benefit for men and none for women or for both men and women [1, 2, 4, 9]. The results may be dissimilar in some cases if different mechanisms are operating to link widowhood and health outcomes across contexts. When comparing India and high-income countries like the United States, the United Kingdom, and the Netherlands, there are significant differences in gender norms, economic mobility, marriage traditions, inheritance traditions, and the extent to which government takes care of certain groups within its citizenry (e.g. the widowed, the aged, the poor, etc.). Our result indicating no relationship between psychological distress and marital status for men was the opposite of the results from a study of older adults from Korea [35]. The findings may between these two countries due to potential differences in gender norms, treatment of wives, and response to the loss of social support.

An important limitation of this study was our inability to examine objective markers of health and disease. Therefore, there are likely undiagnosed cases of mental distress and chronic disease in our sample. Moreover, we were unable to adjust for pre-widowhood disease status, which is likely very important for assessing determinants of health outcomes after widowhood [27]. In addition, this study does not capture the effect of widowhood and widowhood duration on overall health as indicated by mortality. Critical to acknowledge in the interpretation of these results is that the widowed may be more likely to die than non-widowed. It could be that the men in this study are overall a much healthier population than they would be if the widowed men who had already died were still alive. Although the same could be said of the women, men are more likely to die earlier. Thus, this issue might more strongly bias the findings about men than the findings about women. Finally, given the cross-sectional nature of the data, we cannot infer causality from our associational estimates. Future studies may clarify the relevance of marital status to health outcomes among older widowed men and women in India by collecting longitudinal data and biomarkers as well as information on the quality of marital relationships and information about gender norms and sex roles within the household.

## Conclusions

This is the first study to our knowledge that reports on the association between widowhood and several mental and physical-related health outcomes, as well as self-rated health, among a large sample of older adults across India, while adjusting for many demographic and socioeconomic characteristics. Our study suggests important gender differences in how widowhood is associated with self-rated health, psychological distress, hypertension, and diabetes among older adults in India with recent and long-term widowhood predicting worse health for women, but not for men. Incorporating information about marital relationships into the design of intervention programs may help better target potential beneficiaries among older adults in India.

## Additional file

Additional file 1: Multilevel linear and logistic regression that estimates the relationships between explanatory variables and outcomes. (XLSX 56 kb)

## Abbreviations

AOR: Adjusted odds-ratio
CI: Confidence interval
PSU: Primary sampling unit
